# Supplementary material for: Functional annotation of the Hippo pathway somatic mutations in human cancers
Source: Nat Commun. 2024 Nov 21;15:10106. doi: 10.1038/s41467-024-54480-y (PMC11582751; doi:10.1038/s41467-024-54480-y)

Supplementary Figure 1

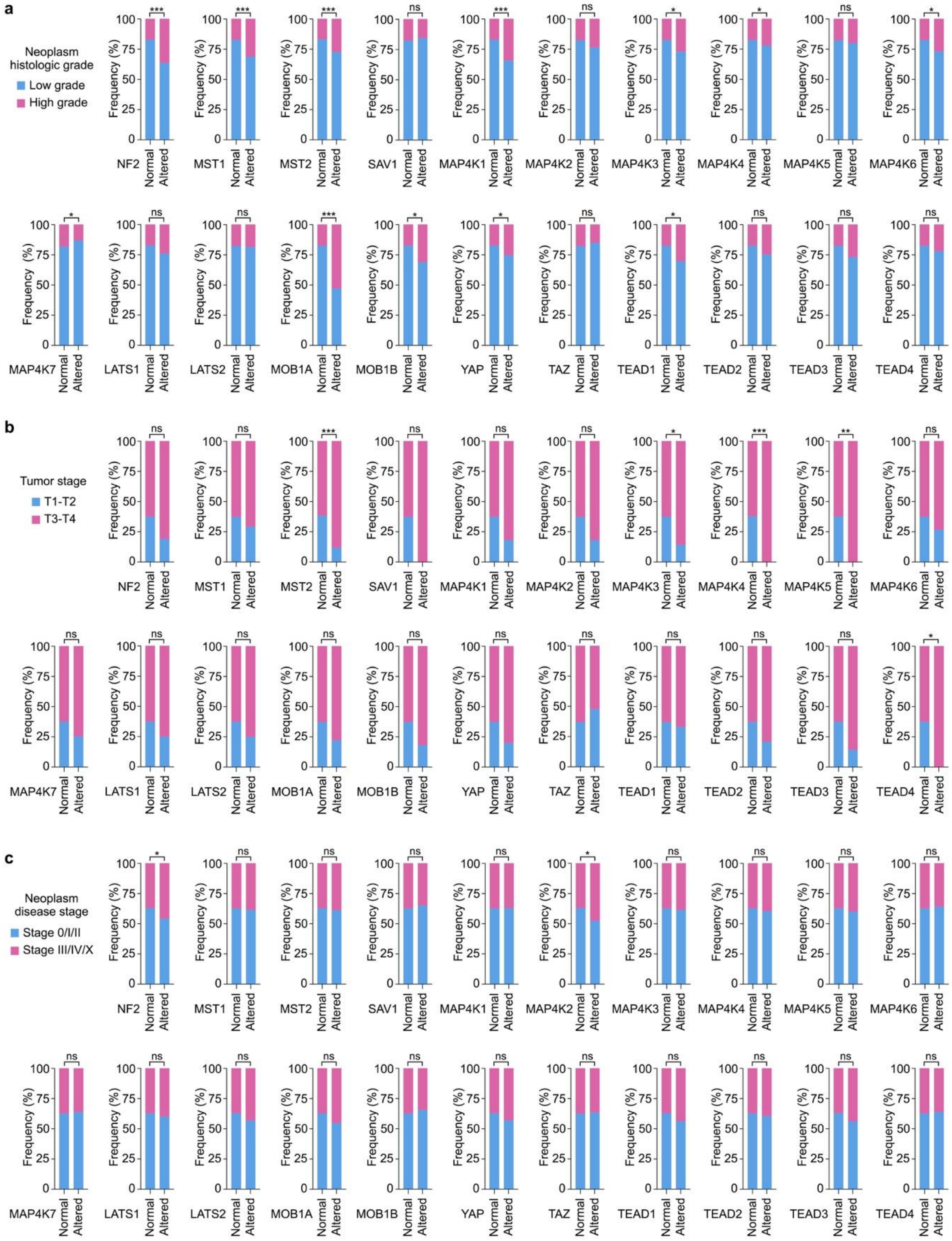

Supplementary Figure 1. Histological analysis of cancer patient samples with individual altered Hippo signaling genes.

(**a-c**) Tumor samples with normal and altered Hippo signaling genes are analyzed for their neoplasm histologic grade (**a**), tumor stage (**b**), and neoplasm disease stage (**c**). ns, no significance. * $p < 0.05$, ** $p < 0.01$, *** $p < 0.001$ (Chi-squared test). Source data are provided as a Source Data file.

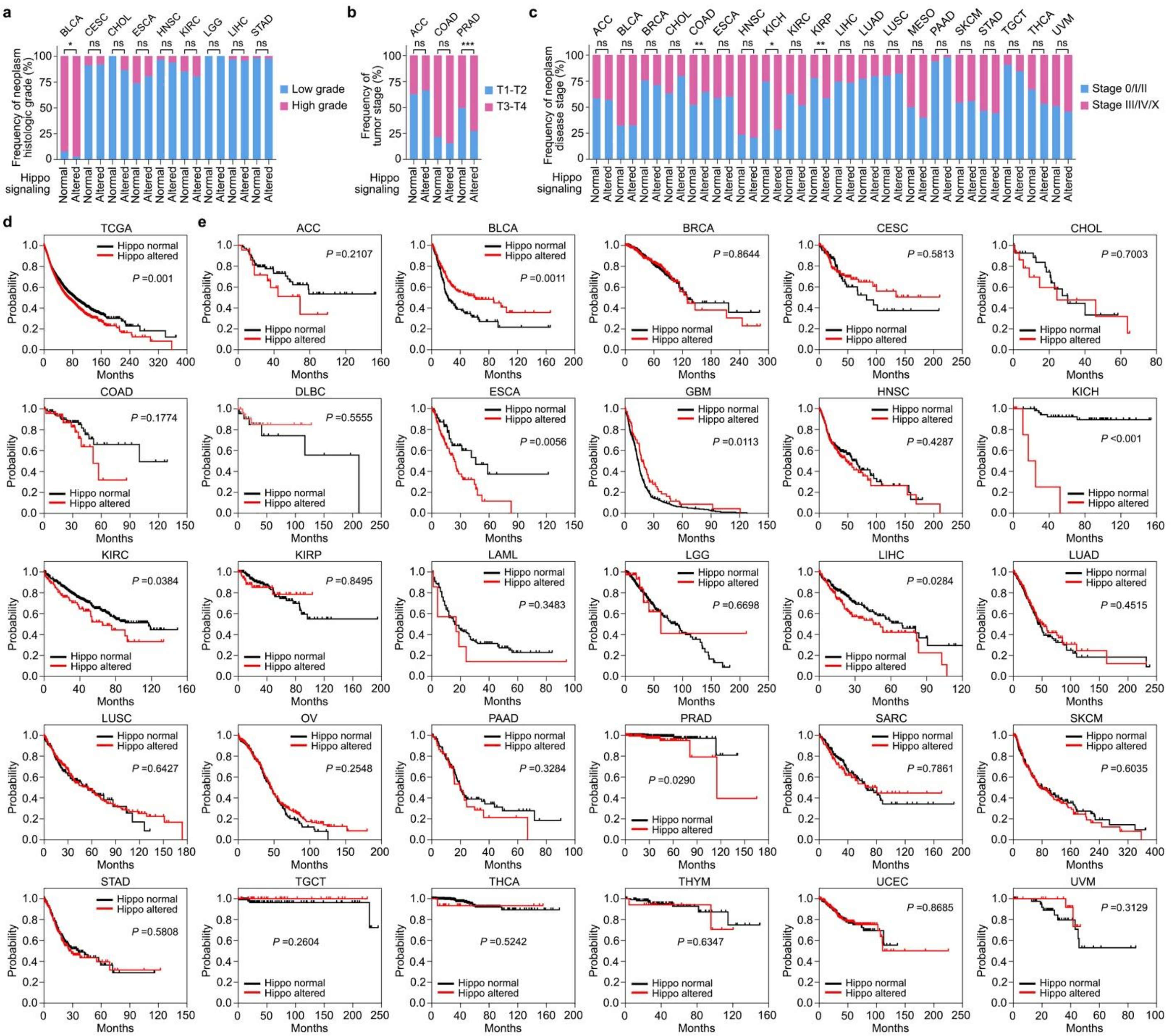
a
b
c
d
e
Frequency of neoplasm histologic grade (%)
Low grade
High grade
Frequency of tumor stage (%)
T1-T2
T3-T4
Frequency of neoplasm disease stage (%)
Stage 0/I/II
Stage III/IV/X
Hippo signaling
Normal
Altered
TCGA
Hippo normal
Hippo altered
Probability
Months
P =0.001
ACC P =0.2107
BLCA P =0.0011
BRCA P =0.8644
CESC P =0.5813
CHOL P =0.7003
COAD P =0.1774
DLBC P =0.5555
ESCA P =0.0056
GBM P =0.0113
HNSC P =0.4287
KICH P <0.001
KIRC P =0.0384
KIRP P =0.8495
LAML P =0.3483
LGG P =0.6698
LIHC P =0.0284
LUAD P =0.4515
LUSC P =0.6427
OV P =0.2548
PAAD P =0.3284
PRAD P =0.0290
SARC P =0.7861
SKCM P =0.6035
STAD P =0.5808
TGCT P =0.2604
THCA P =0.5242
THYM P =0.6347
UCEC P =0.8685
UVM P =0.3129

Supplementary Figure 2. Histological and survival analyses of cancer patients with altered Hippo signaling genes in different cancer types.

(**a-c**) Tumor samples with normal and altered Hippo signaling genes are analyzed for their neoplasm histologic grade (**a**), tumor stage (**b**), and neoplasm disease stage (**c**) in different cancer types. ns, no significance. * $p < 0.05$, ** $p < 0.01$, *** $p < 0.001$ (Chi-squared test).

(**d-e**) Overall survival analysis of cancer patients with normal and altered Hippo signaling genes. The overall survival of cancer patients with normal and altered Hippo signaling genes in TCGA (**d**) and the indicated cancer types (**e**) was analyzed using log-rank (Mantel-Cox) test.

Source data are provided as a Source Data file.

Supplementary Figure 3

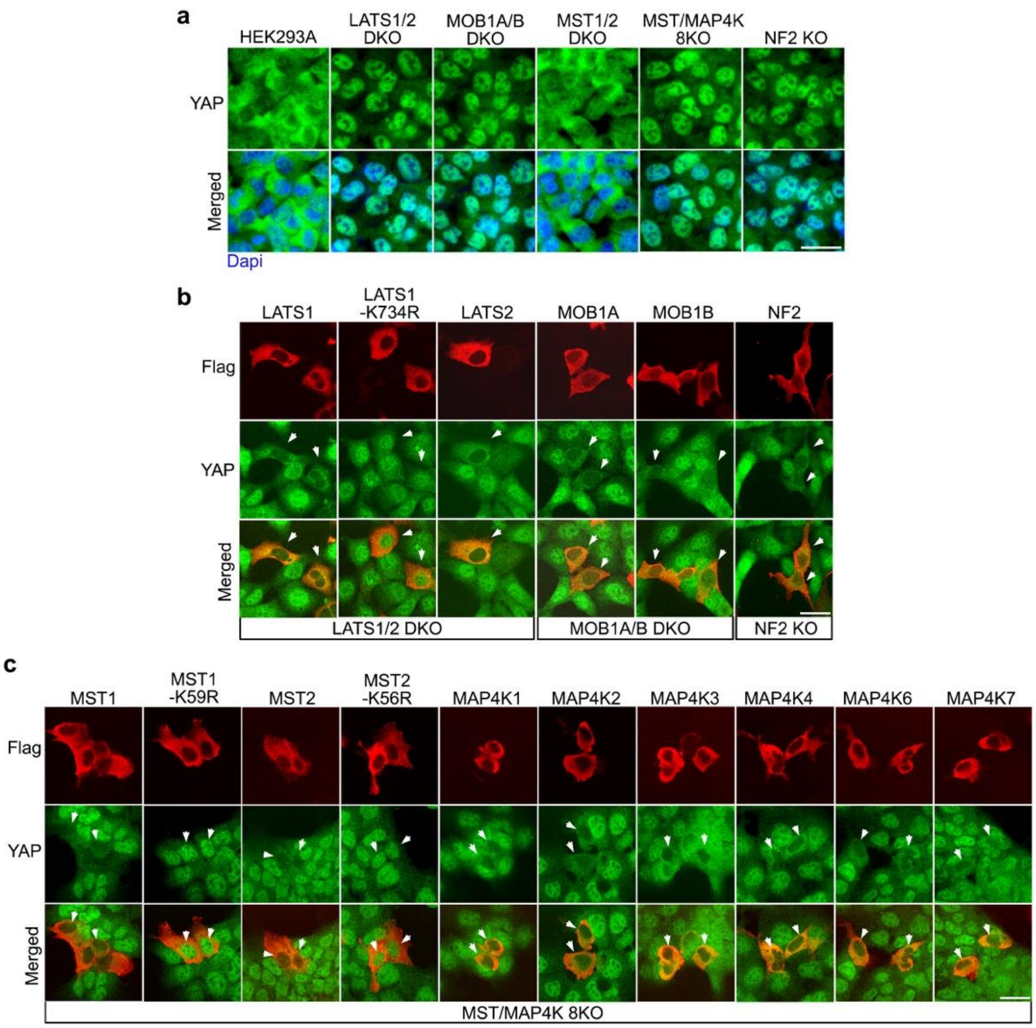

Supplementary Figure 3. Validation of Hippo pathway gene knockout (KO) cells for missense mutation screen.

(**a**) YAP is enriched the nucleus of the LATS1/2 double KO (DKO), MOB1A/B DKO, MST/MAP4K-8KO, and NF2 KO HEK293A cells. The indicated Hippo component KO HEK293A cells were subjected to immunofluorescent staining. Nucleus was visualized by Dapi. Scale bar, 20 μm.

(**b-c**) Validation of the Hippo component KO HEK293A cells. The indicated LATS1/2 DKO (**b**), MOB1A/B DKO (**b**), NF2 KO (**b**), and MST/MAP4K-8KO (**c**) HEK293A cells were transfected with the indicated constructs and subjected to immunofluorescent staining. Scale bar, 30 μm. Arrows showed the cells expressing the indicated constructs.

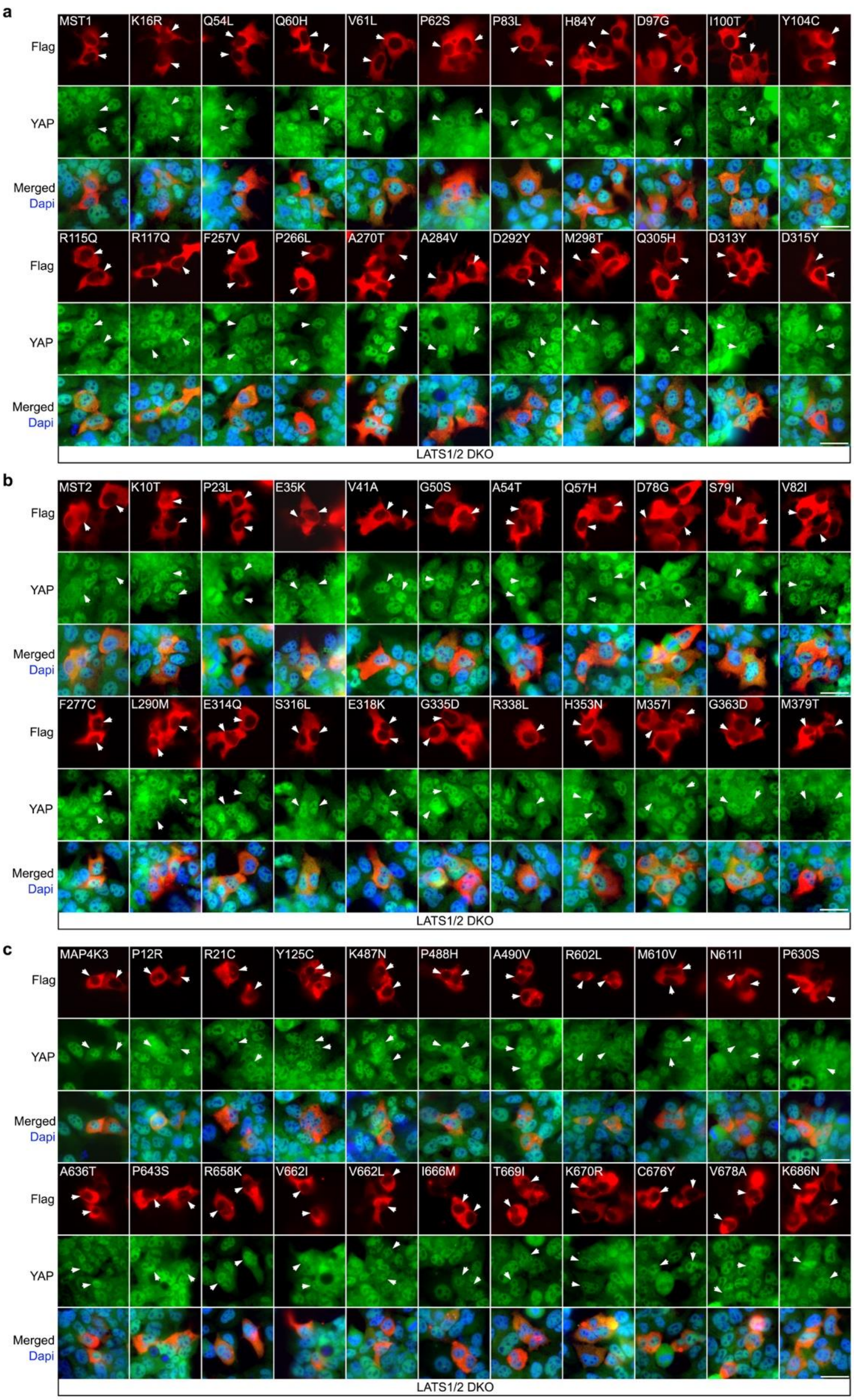
a
Flag
YAP
Merged
Dapi
MST1
K16R
Q54L
Q60H
V61L
P62S
P83L
H84Y
D97G
I100T
Y104C
R115Q
R117Q
F257V
P266L
A270T
A284V
D292Y
M298T
Q305H
D313Y
D315Y
LATS1/2 DKO
b
MST2
K10T
P23L
E35K
V41A
G50S
A54T
Q57H
D78G
S79I
V82I
F277C
L290M
E314Q
S316L
E318K
G335D
R338L
H353N
M357I
G363D
M379T
LATS1/2 DKO
c
MAP4K3
P12R
R21C
Y125C
K487N
P488H
A490V
R602L
M610V
N611I
P630S
A636T
P643S
R658K
V662I
V662L
I666M
T669I
K670R
C676Y
V678A
K686N
LATS1/2 DKO

Supplementary Figure 4. Characterization of the neutral mutations of MST1, MST2 and MAP4K3 in the LATS1/2 DKO cells.

(**a-c**) The LATS1/2 DKO HEK293A cells were transfected with the indicated neutral missense mutants of MST1 (**a**), MST2 (**b**), and MAP4K3 (**c**) and subjected to immunofluorescent staining. Scale bar, 30 μm. Arrows showed the cells expressing the indicated constructs.

Supplementary Figure 5

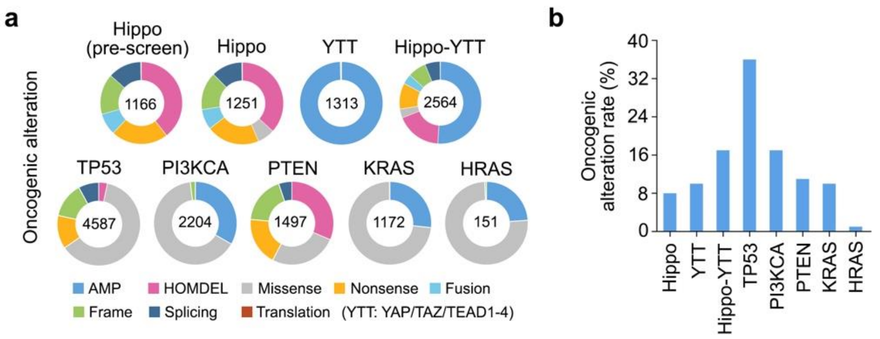

Supplementary Figure 5. Analysis of the Hippo signaling oncogenic alterations.

(**a**) The oncogenic alterations of Hippo signaling genes and the indicated tumor suppressor genes and oncogenes were summarized. The number of affected cancer patients was indicated.

(**b**) The oncogenic alteration rates for Hippo signaling genes and the indicated cancer-related genes were shown.

Source data are provided as a Source Data file.

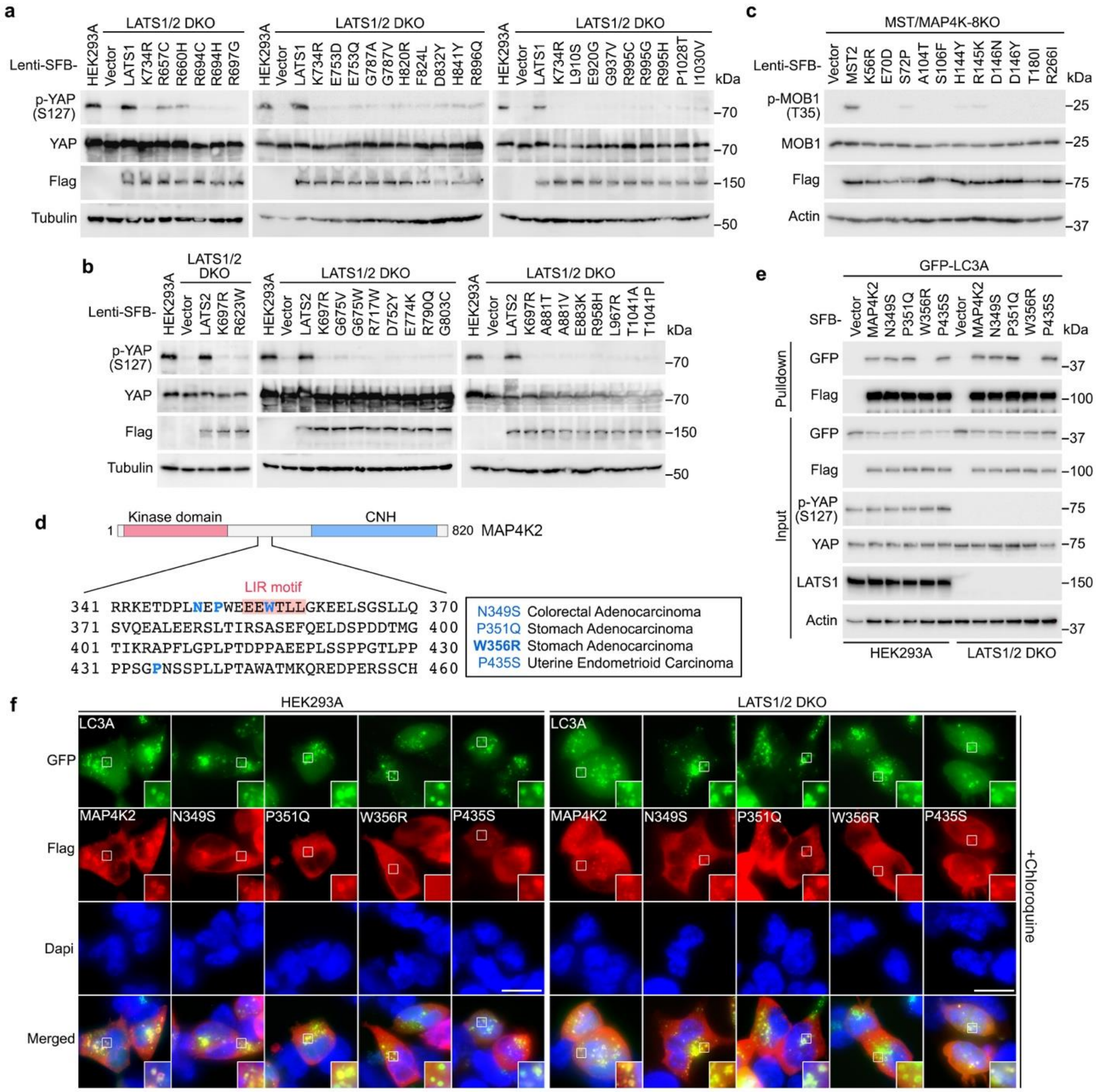
a
LATS1/2 DKO
Lenti-SFB-
HEK293A
Vector
LATS1
K734R
R657C
R660H
R694C
R694H
R697G
E753D
E753Q
G787A
G787V
H820R
F824L
D832Y
H841Y
R896Q
L910S
E920G
G937V
R995C
R995G
R995H
P1028T
I1030V
kDa
p-YAP (S127)
YAP
Flag
Tubulin
b
LATS2
K697R
R623W
G675V
G675W
R717W
D752Y
E774K
R790Q
G803C
A881T
A881V
E883K
R958H
L967R
T1041A
T1041P
c
MST/MAP4K-8KO
MST2
K56R
E70D
S72P
A104T
S106F
H144Y
R145K
D146N
D146Y
T180I
R266I
p-MOB1 (T35)
MOB1
Actin
d
Kinase domain
CNH
1
820
MAP4K2
LIR motif
341 RRKETDPLNEPWEEEWTLLGKEELSGSLLQ 370
371 SVQEALEERSLTIRSASEFQELDSPDDTMG 400
401 TIKRAPFLGPLPTDPPAEEPLSSPPGTLPP 430
431 PPSGPNSSPLLPTAWATMKQREDPERSSCH 460
N349S Colorectal Adenocarcinoma
P351Q Stomach Adenocarcinoma
W356R Stomach Adenocarcinoma
P435S Uterine Endometrioid Carcinoma
e
GFP-LC3A
SFB-
MAP4K2
N349S
P351Q
W356R
P435S
Pulldown
Input
GFP
LATS1
HEK293A
LATS1/2 DKO
f
LC3A
Dapi
Merged
+Chloroquine

Supplementary Figure 6. Characterization of the Hippo LOF and neutral missense mutations.

(**a-b**) Analysis of LATS1/2 LOF missense mutations in stable cells. The LATS1/2 DKO HEK293A cells stably expressing the indicated LOF mutants of LATS1 (**a**) and LATS2 (**b**) were subjected to Western blot. A representative blot of two independent experiments is shown.

(**c**) Analysis of MST2 LOF missense mutations in stable cells. The MST/MAP4K-8KO HEK293A cells stably expressing the indicated MST2 LOF mutants were subjected to Western blot. A representative blot of two independent experiments is shown.

(**d**) The MAP4K2 neutral mutation W356R is identified in its LIR motif. W356R and its nearby neutral mutations were indicated in blue. The LIR motif was highlighted in red.

(**e**) The W356R neutral mutation disrupted the interaction of MAP4K2 with LC3A in both wild-type and LATS1/2 DKO HEK293A cells. Wild-type and LATS1/2 DKO HEK293A cells were transfected with GFP-LC3A and the indicated SFB-MAP4K2 constructs and subjected to pulldown assay using S protein beads. A representative blot of two independent experiments is shown.

(**f**) The W356R neutral mutation disrupted the co-localization of MAP4K2 with LC3A in chloroquine-treated wild-type and LATS1/2 DKO HEK293A cells. The constructs encoding GFP-LC3A and the indicated SFB-MAP4K2 mutants were co-expressed in wild-type and LATS1/2 DKO HEK293A cells, treated with chloroquine (200 μM) for 4 hours, and subjected to immunofluorescent staining. The indicated regions in the box are shown 3 times enlarged. Scale bar, 20 μm.

Supplementary Figure 7

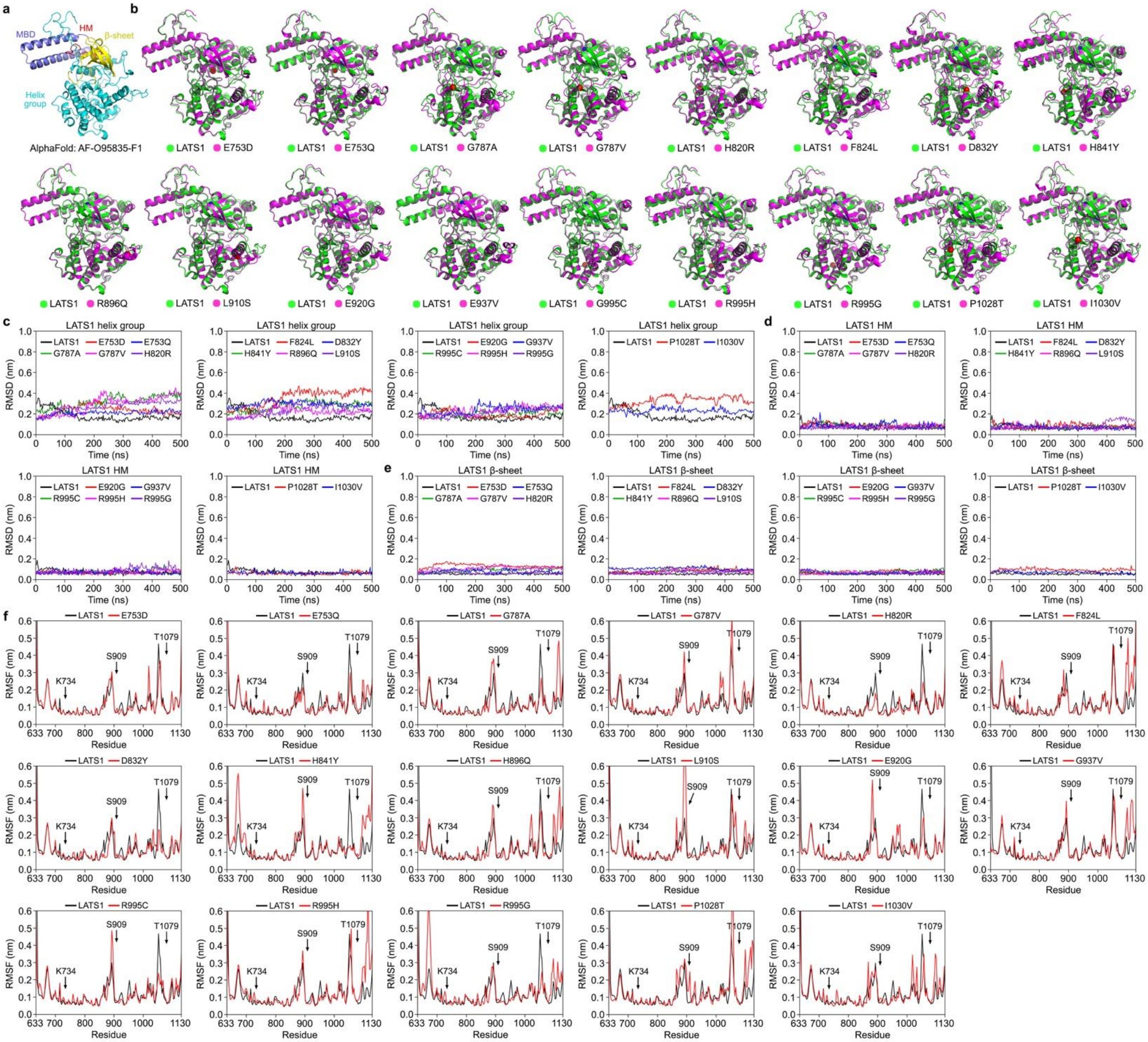

Supplementary Figure 7. Molecular dynamics analysis of the LATS1 LOF missense mutations.

(**a**) The LATS1 protein structure including its MOB1-binding domain (MBD) and kinase domain (predicted using AlphaFold2) is used for the molecular dynamics analysis. The MBD, helix group, hydrophobic motif (HM), and β-sheet were shown in different colors.

(**b**) The LATS1 LOF missense mutations induce its protein conformational change. The overlay structures were shown for LATS1 and its indicated LOF missense mutants.

(**c-e**) RMSD analysis is performed to examine the LATS1 LOF missense mutations-caused effects on its kinase domain structure, where LATS1 helix group (**c**), HM (**d**), and β-sheet (**e**) were examined.

(**f**) RMSF analysis is performed for the LATS1 kinase domain LOF missense mutants. LATS1 ATP-binding site K734, autophosphorylation site S909, and HM phosphorylation site T1079 were indicated.

Supplementary Figure 8

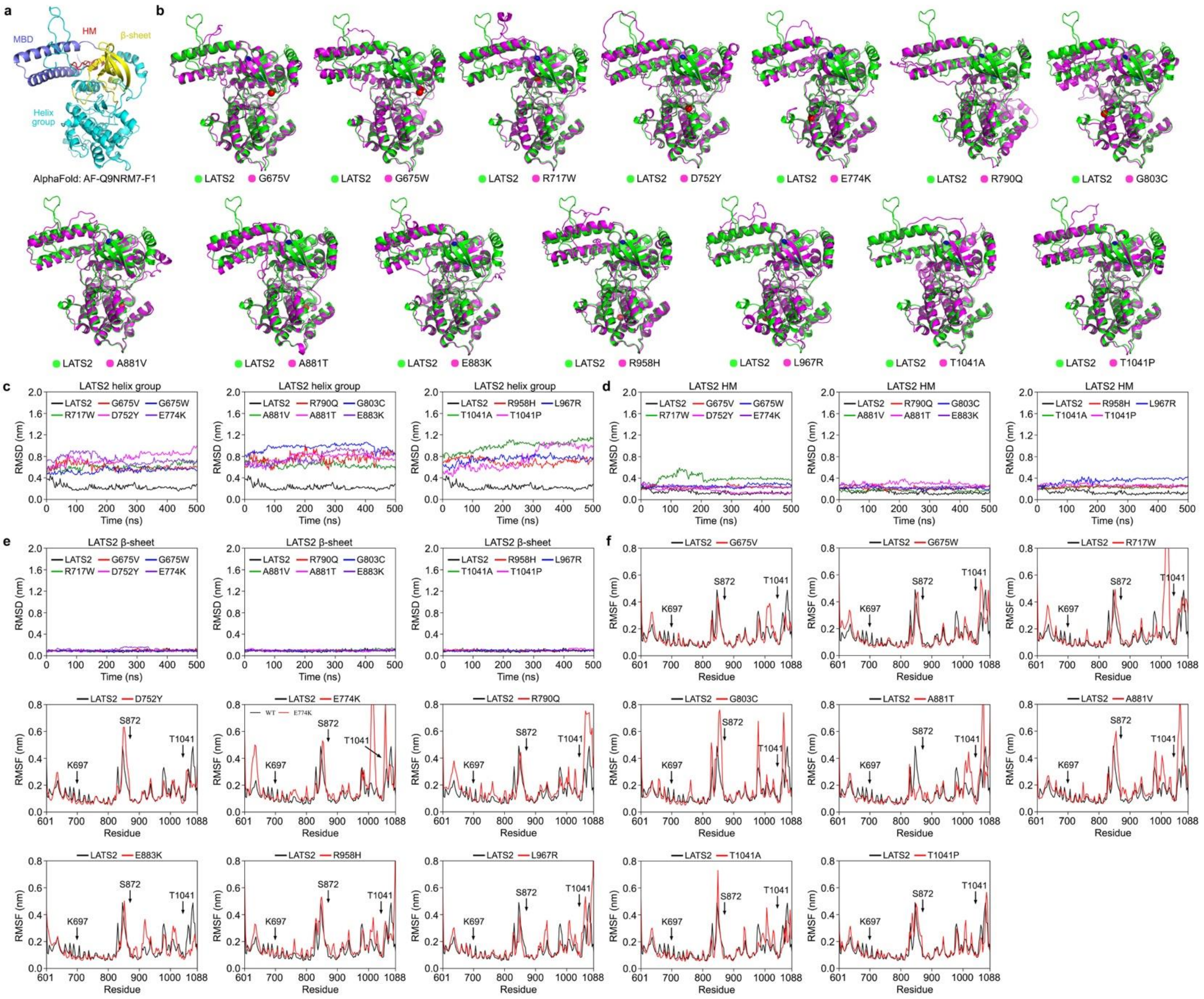

Supplementary Figure 8. Molecular dynamics analysis of the LATS2 LOF missense mutations.

(**a**) The LATS2 protein structure including its MOB1-binding domain (MBD) and kinase domain (predicted using AlphaFold2) is used for the molecular dynamics analysis. The MBD, helix group, hydrophobic motif (HM), and β-sheet were shown in different colors.

(**b**) The LATS2 LOF missense mutations induce its protein conformational change. The overlay structures were shown for LATS2 and its indicated LOF missense mutants.

(**c-e**) RMSD analysis is performed to examine the LATS2 LOF missense mutations-caused effects on its kinase domain protein structure, where LATS2 helix group (**c**), HM (**d**), and β-sheet (**e**) were examined.

(**f**) RMSF analysis is performed for the LATS2 kinase domain LOF missense mutants. LATS2 ATP-binding site K697, autophosphorylation site S872, and HM phosphorylation site T1041 were indicated.

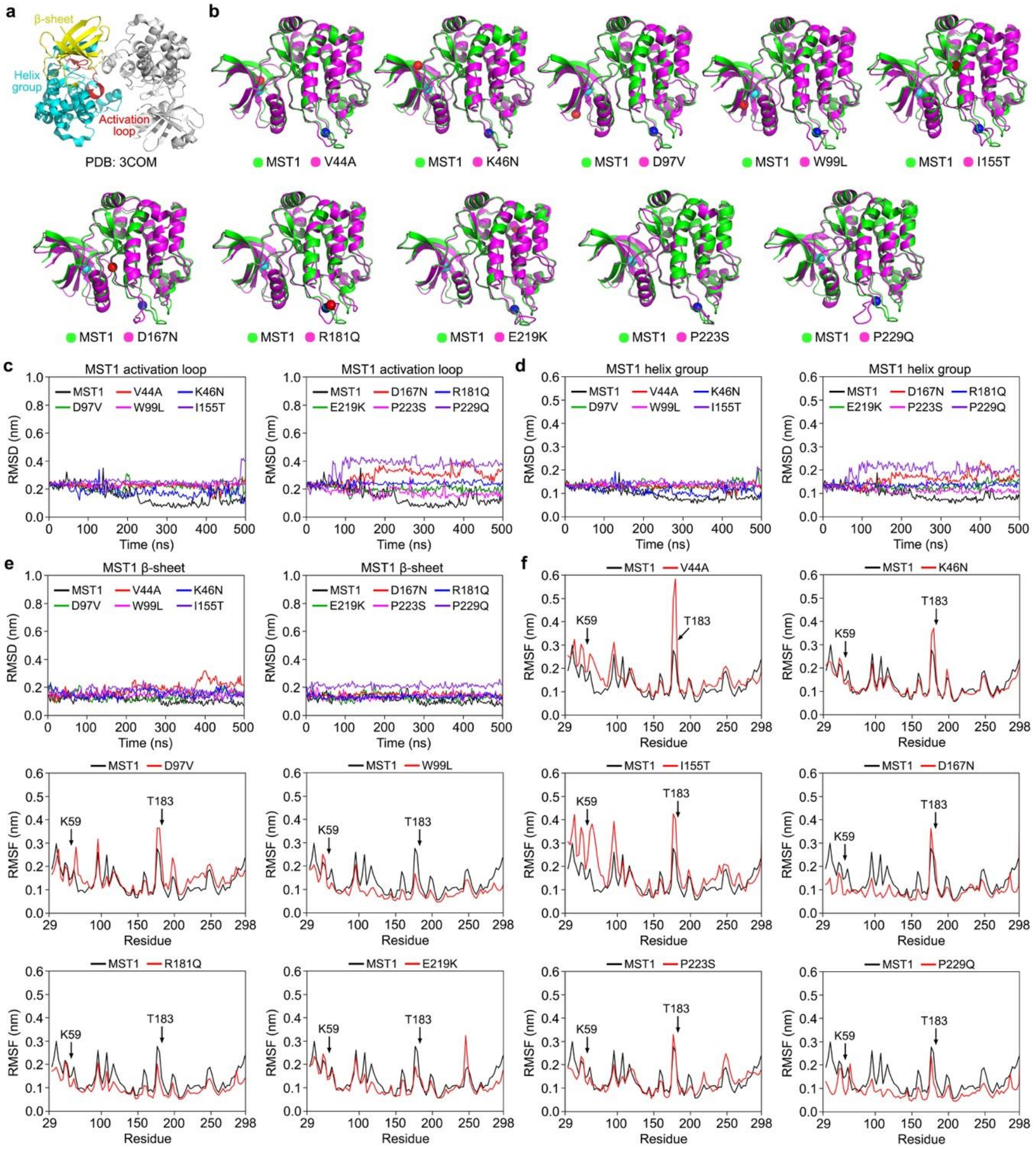
a
β-sheet
Helix group
Activation loop
PDB: 3COM
b
MST1 V44A
MST1 K46N
MST1 D97V
MST1 W99L
MST1 I155T
MST1 D167N
MST1 R181Q
MST1 E219K
MST1 P223S
MST1 P229Q
c
MST1 activation loop
d
MST1 helix group
e
MST1 β-sheet
RMSD (nm)
Time (ns)
f
RMSF (nm)
Residue
K59
T183

Supplementary Figure 9. Molecular dynamics analysis of the MST1 LOF missense mutations.

(**a**) The MST1 kinase domain protein structure (PDB: 3COM) is used for the molecular dynamics analysis. The activation loop, helix group, and β-sheet were shown in different colors.

(**b**) The MST1 LOF missense mutations induce its kinase domain conformational change. The kinase domain overlay structures were shown for MST1 and its LOF missense mutants.

(**c-e**) RMSD analysis is performed to examine the MST1 LOF missense mutations-caused effects on its kinase domain protein structure, where MST1 kinase domain activation loop (**c**), helix group (**d**), and β-sheet (**e**) were examined.

(**f**) RMSF analysis is performed for the MST1 kinase domain LOF missense mutants. MST1 ATP-binding site K59 and autophosphorylation site T183 were indicated.

Supplementary Figure 10

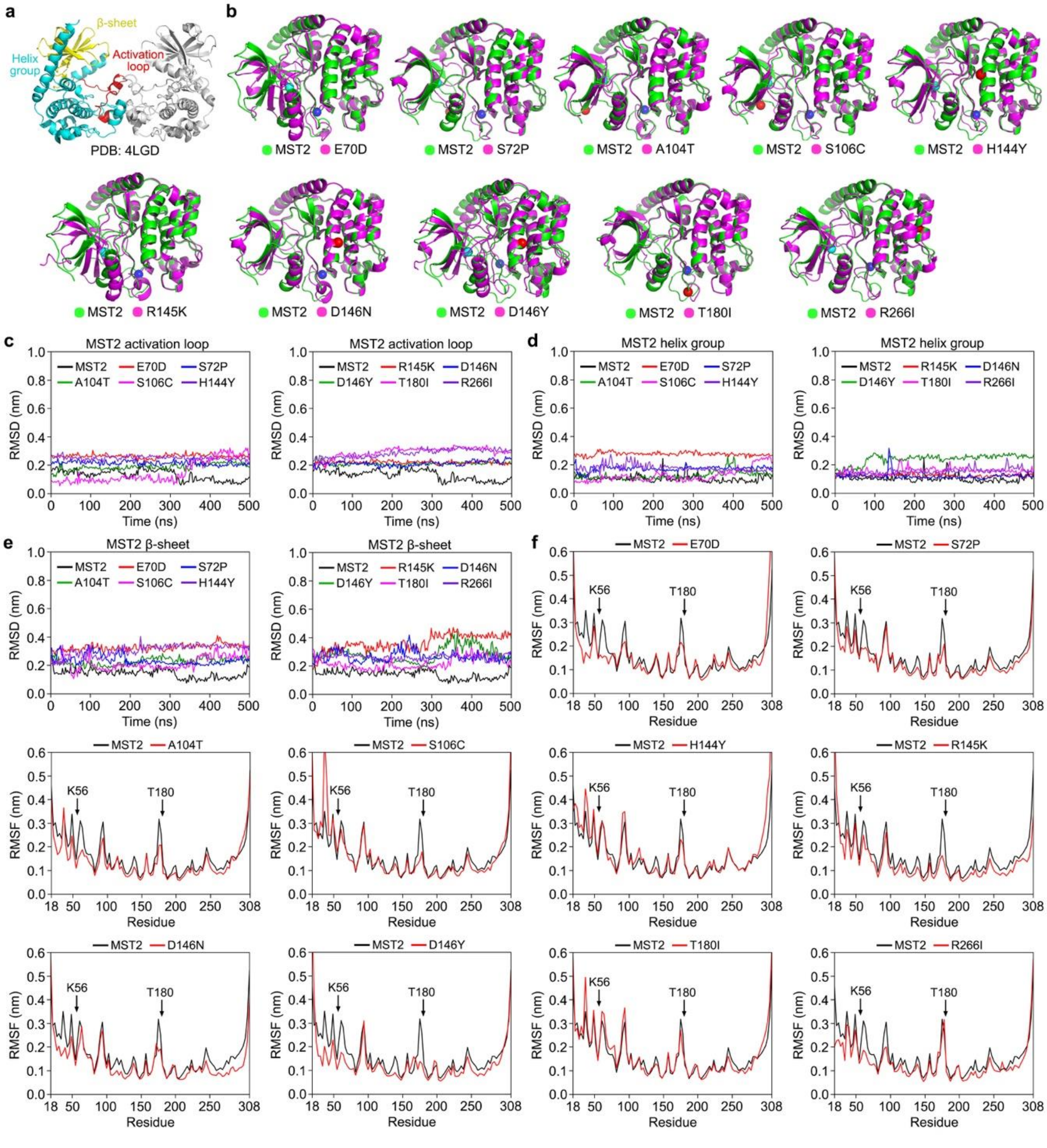

Supplementary Figure 10. Molecular dynamics analysis of the MST2 LOF missense mutations.

(**a**) The MST2 kinase domain protein structure (PDB: 4LGD) is used for the molecular dynamics analysis. The activation loop, helix group, and β-sheet were shown in different colors.

(**b**) The MST2 LOF missense mutations induce its kinase domain conformational change. The kinase domain overlay structures were shown for MST2 and its indicated LOF missense mutants.

(**c-e**) RMSD analysis is performed to examine the MST2 LOF missense mutations-caused effects on its kinase domain protein structure, where MST2 kinase domain activation loop (**c**), helix group (**d**), and β-sheet (**e**) were examined.

(**f**) RMSF analysis is performed for the MST2 kinase domain LOF missense mutants. MST2 ATP-binding site K56 and autophosphorylation site T180 were indicated.

Supplementary Figure 11

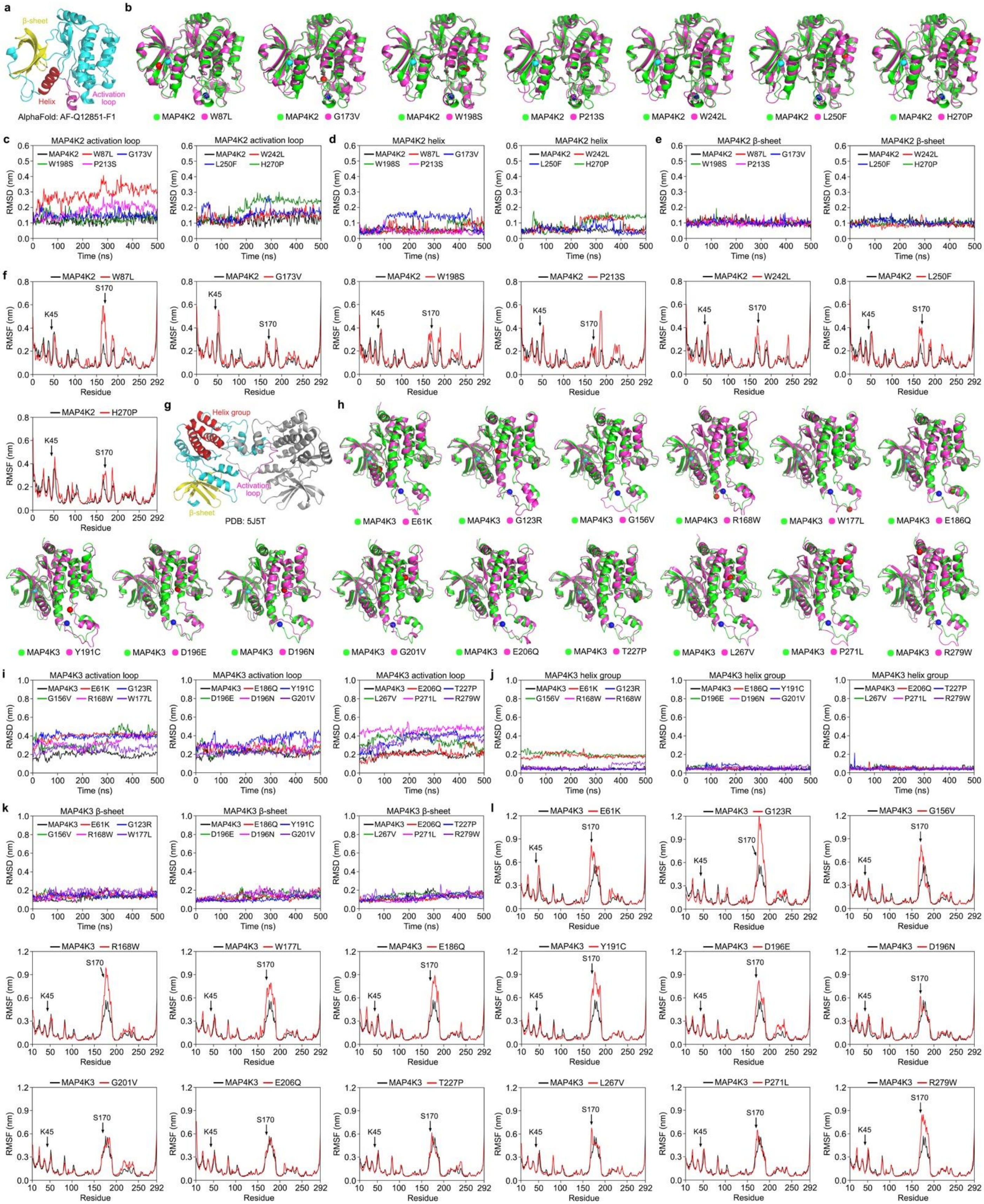

Supplementary Figure 11. Molecular dynamics analysis of the MAP4K2/3 LOF missense mutations.

(**a**) The MAP4K2 kinase domain protein structure (predicted using AlphaFold2) is used for the molecular dynamics analysis. The activation loop, helix region, and β-sheet were shown in different colors.

(**b**) The MAP4K2 LOF missense mutations induce its kinase domain conformational change. The kinase domain overlay structures were shown for MAP4K2 and its indicated LOF missense mutants.

(**c-e**) RMSD analysis is performed to examine the MAP4K2 LOF missense mutations-caused effects on its kinase domain protein structure, where MAP4K2 kinase domain activation loop (**c**), helix group (**d**), and β-sheet (**e**) were examined.

(**f**) RMSF analysis is performed for the MAP4K2 kinase domain LOF missense mutants. MAP4K2 ATP-binding site K45 and autophosphorylation site S170 were indicated.

(**g**) The MAP4K3 kinase domain protein structure (PDB: 5J5T) is used for the molecular dynamics analysis. The activation loop, helix group, and β-sheet were shown in different colors.

(**h**) The MAP4K3 LOF missense mutations induce its kinase domain conformational change. The kinase domain overlay structures were shown for MAP4K3 and its indicated LOF missense mutants.

(**i-k**) RMSD analysis is performed to examine the MAP4K3 LOF missense mutations-caused effects on its kinase domain protein structure, where MAP4K3 kinase domain activation loop (**i**), helix group (**j**), and β-sheet (**k**) were examined.

(**l**) RMSF analysis is performed for the MAP4K3 kinase domain LOF missense mutants. MAP4K3 ATP-binding site K45 and autophosphorylation site S170 were indicated.

Supplementary Figure 12

a

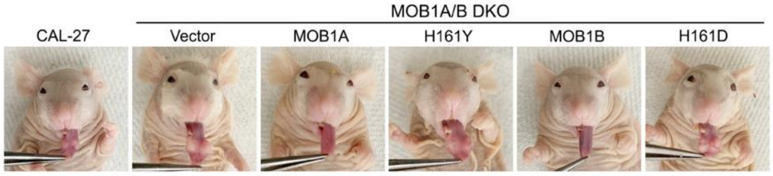

b

Bait: MOB1A		Bait: MOB1A-H161Y		Bait: MOB1B		Bait: MOB1B-H161D	
9/166	**MOB1A**	9/114	**MOB1A**	10/286	**MOB1B**	8/45	**MOB1B**
22/36	**STK38L**	8/56	UBA52	32/82	**STK38L**	20/27	HSPA8
28/32	TCOF1	33/37	TCOF1	37/71	**LATS1**	22/24	CKAP4
23/25	**LATS1**	23/28	RPN1	32/39	TCOF1	42/46	ACACA
21/24	SRRM2	23/25	SRRM2	20/23	HSPA5	6/22	UBA52
15/19	EIF5B	18/21	HSPA5	20/22	SRRM2	15/18	HSPA1B
16/17	HSPA5	18/20	CKAP4	14/17	HSPA8	16/17	HSPA5
5/17	UBA52	13/19	SLC3A2	13/17	EIF5B	14/17	SLC3A2
15/16	HSPA8	15/17	RRBP1	15/16	ACACA	11/16	TUBA1C
13/13	RRBP1	13/15	HSPA8	6/16	UBA52	14/14	HECTD1
13/13	ACACA	12/15	EIF5B	10/10	RRBP1	11/13	HSPA1L
11/11	SLC3A2	12/14	HSPD1	10/10	CKAP4	12/12	RPN1
6/7	**STK38**	13/13	ACACA	9/10	**STK38**	11/11	TOCF1
6/6	SRPRB	9/11	SRPRB	9/9	SLC3A2	10/10	HSPD1
6/6	HSPA2	6/6	ACTA2	6/8	**LATS2**	9/10	EEF1A1
2/2	**LATS2**	6/6	HSPA2	7/7	RPN1	7/9	HSPB1
2/2	PSMA8	1/2	PSMA8	7/7	HSPA9	7/7	SRPRB
Unique/Total	Prey	Unique/Total	Prey	Unique/Total	Prey	Unique/Total	Prey

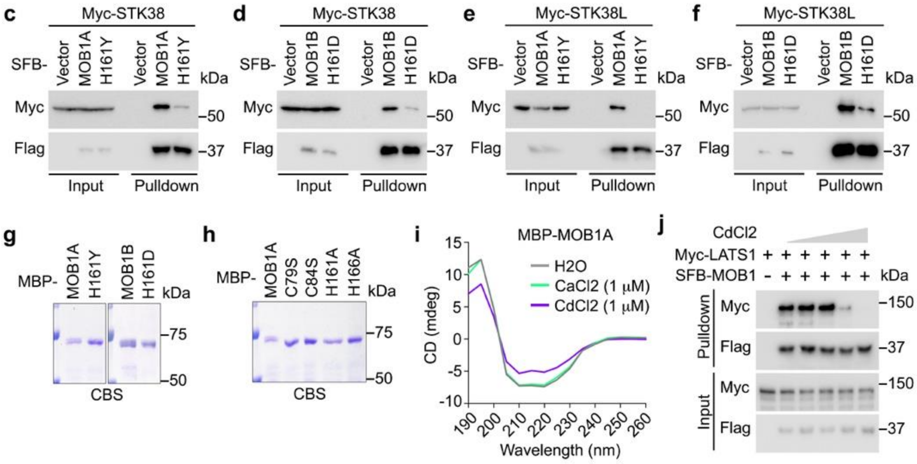

Supplementary Figure 12. The MOB1A/B LOF missense mutations interfere with MOB1A/B ZNF domains and protein functions.

(**a**) The MOB1A/B LOF missense mutants promote CAL-27 xenograft tumor growth in mouse tongues. The representative images of mice and their tongues were shown.

(**b**) TAP-MS analysis of the protein-protein interaction network of MOB1A/B and their LOF missense mutants. The numbers of unique and total peptides for each identified protein were shown.

(**c-f**) The MOB1A/B LOF missense mutations disrupt the interaction of MOB1A/B with STK38 and STK38L. HEK293T cells were transfected with Myc-STK38/L and the indicated SFB-MOB1A/B constructs and subjected to pulldown assay using S protein beads. A representative blot of two independent experiments is shown.

(**g-h**) The indicated MBP-tagged MOB1A/B proteins are purified from bacteria and visualized by Coomassie blue staining (CBS).

(**i**) MOB1A protein structure is changed upon Cd treatment. The MBP-tagged MOB1A protein was incubated with 1 μM CaCl2 or CdCl2 and subjected to circular dichroism analysis.

(**j**) Cd treatment targets the MOB1-LATS1 complex formation. HEK293T cells were transfected with Myc-LATS1 and SFB-MOB1A constructs. Cell lysates were incubated with CdCl2 at different concentrations (i.e., 0 μM, 1 μM, 2.5 μM, 5 μM, and 10 μM) and subjected to pulldown assay. A representative blot of two independent experiments is shown.

Source data are provided as a Source Data file.

Supplementary Figure 13

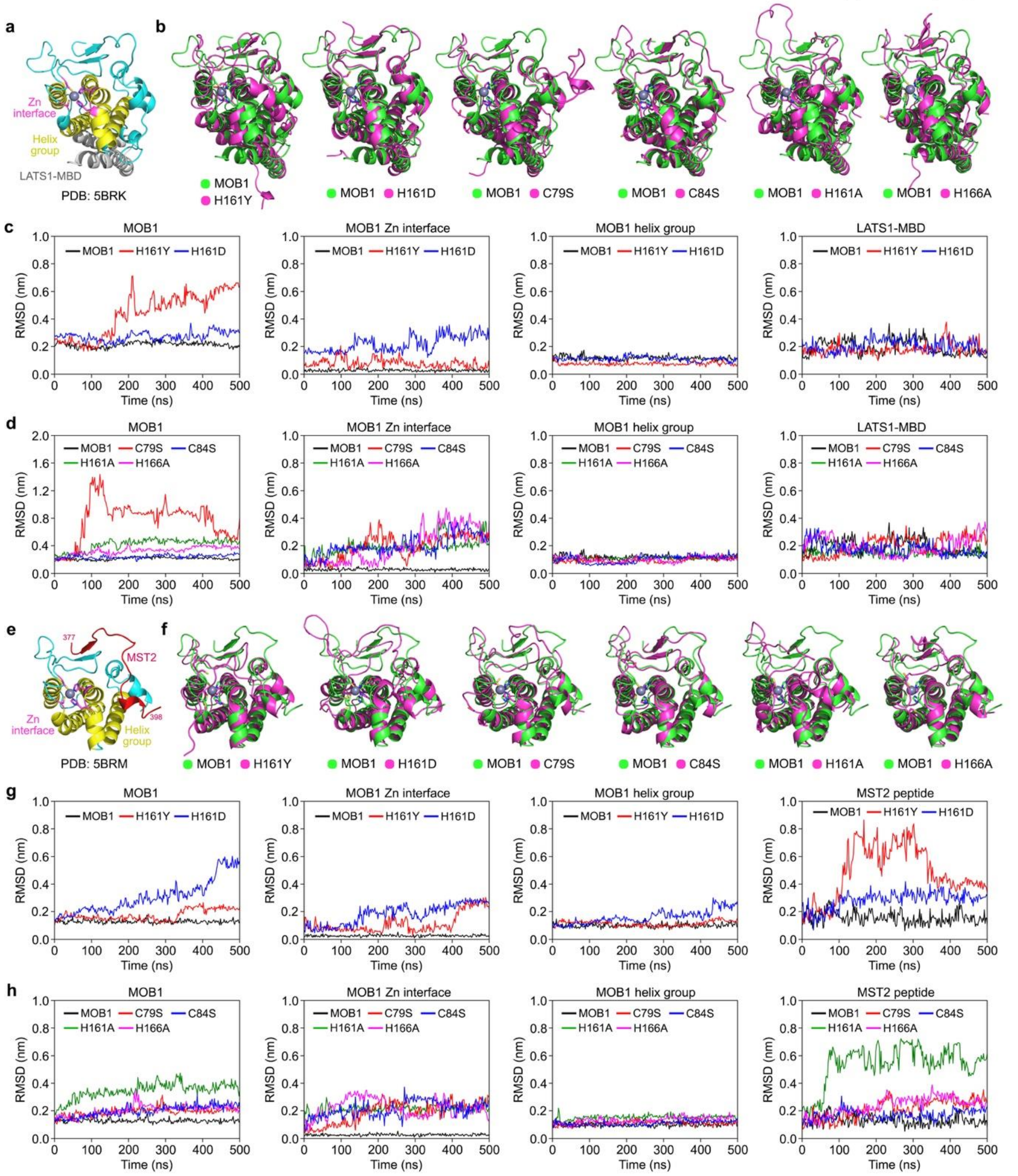

Supplementary Figure 13. Molecular dynamics analysis of the MOB1 LOF missense mutations.

(**a**) The p-MOB1/LATS1-MBD co-crystal structure (PDB: 5BRK) is used for the molecular dynamics analysis. The Zn interface and helix group of MOB1 and LATS1-MBD were shown in different colors.

(**b**) The MOB1 LOF and ZNF domain mutations induce the conformational change of the p-MOB1/LATS1-MBD complex. The p-MOB1/LATS1-MBD overlay structures were shown for MOB1 and its indicated LOF and ZNF domain mutants.

(**c-d**) RMSD analysis is performed to examine the MOB1 LOF missense mutations (**c**) and its ZNF domain mutations (**d**)-caused effects on the p-MOB1/LATS1-MBD co-crystal structure. RMSD analysis was performed for overall MOB1, its Zn interface and helix group, and LATS1-MBD within the p-MOB1/LATS1-MBD co-crystal structure.

(**e**) The MOB1/MST2-peptide co-crystal structure (PDB: 5BRM) is used for the molecular dynamics analysis. The Zn interface and helix group of MOB1 and MST2-peptide were shown in different colors.

(**f**) The MOB1 LOF and ZNF domain mutations induce the conformational change of the MOB1/MST2-peptide complex. The MOB1/MST2-peptide overlay structures were shown for MOB1 and its indicated LOF and ZNF domain mutants.

(**g-h**) RMSD analysis is performed to examine the MOB1 LOF missense mutations (**g**) and its ZNF domain mutations (**h**)-caused effects on the MOB1/MST2-peptide co-crystal structure. RMSD analysis was performed for overall MOB1, its Zn interface and helix group, and MST2-peptide within the MOB1/MST2-peptide co-crystal structure.

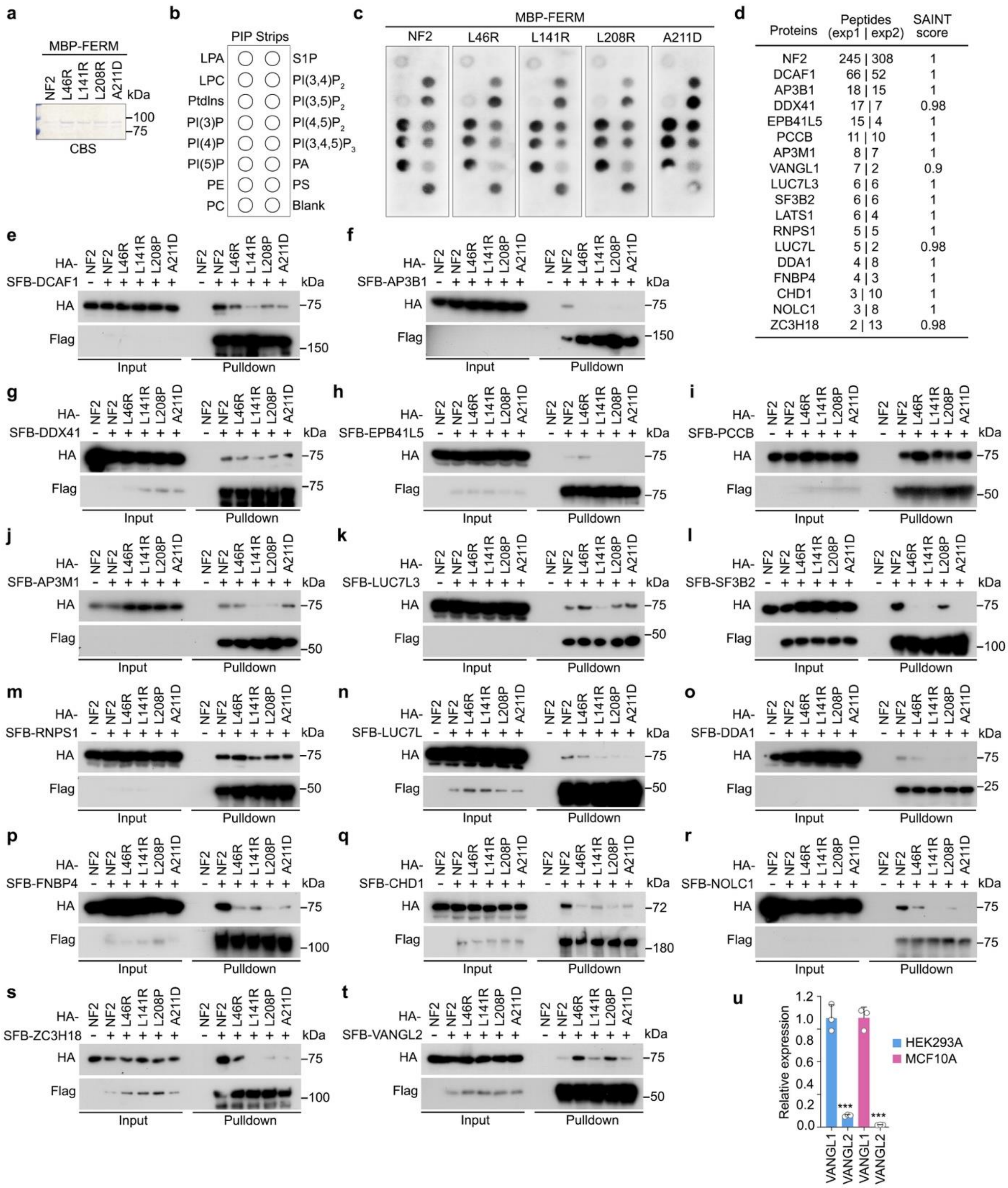

d

Proteins	Peptides (exp1 | exp2)	SAINT score
NF2	245 | 308	1
DCAF1	66 | 52	1
AP3B1	18 | 15	1
DDX41	17 | 7	0.98
EPB41L5	15 | 4	1
PCCB	11 | 10	1
AP3M1	8 | 7	1
VANGL1	7 | 2	0.9
LUC7L3	6 | 6	1
SF3B2	6 | 6	1
LATS1	6 | 4	1
RNPS1	5 | 5	1
LUC7L	5 | 2	0.98
DDA1	4 | 8	1
FNBP4	4 | 3	1
CHD1	3 | 10	1
NOLC1	3 | 8	1
ZC3H18	2 | 13	0.98

Supplementary Figure 14. The NF2 LOF mutations do not affect NF2 lipid-binding ability but promote its interaction with VANGL proteins.

(**a**) The indicated MBP-tagged NF2 FERM proteins are purified from bacteria and visualized by Coomassie blue staining (CBS).

(**b**) Illustration of the PIP Strips membrane used for the protein-lipid interaction study.

(**c**) The NF2 LOF mutations do not affect the interaction of NF2 FERM domain with the phospholipids. The PIP Strips membranes were incubated with the bacterially purified MBP-tagged NF2 FERM domain mutant proteins and subjected to Western blot. A representative blot of two independent experiments is shown.

(**d**) Summary of the NF2 HCIPs identified through two biologically repeated TAP-MS analyses. The total peptide number and SAINT score for each HCIP were shown.

(**e-s**) Characterization of the NF2 LOF mutations-caused effects on the interaction of NF2 with its HCIPs. HEK293T cells were transfected with HA-NF2 LOF mutants and the indicated SFB-tagged HCIPs and subjected to pulldown assay using S protein beads. A representative blot of two independent experiments is shown.

(**t**) The NF2 LOF mutations promote its interaction with VANGL2. HEK293T cells were transfected with HA-NF2 LOF mutants and SFB-VANGL2 and subjected to pulldown assay. A representative blot of two independent experiments is shown.

(**u**) *VANGL2* is lowly expressed in HEK293A and MCF10A cells as compared to *VANGL1*. The transcription of *VANGL1* and *VANGL2* was examined by q-PCR (mean ± s.d., n = 3 biological replicates). *** $p < 0.001$ (two-tailed Student's *t*-test).

Source data are provided as a Source Data file.

Uncropped scans of all blots and gels

Supplementary Figure 6

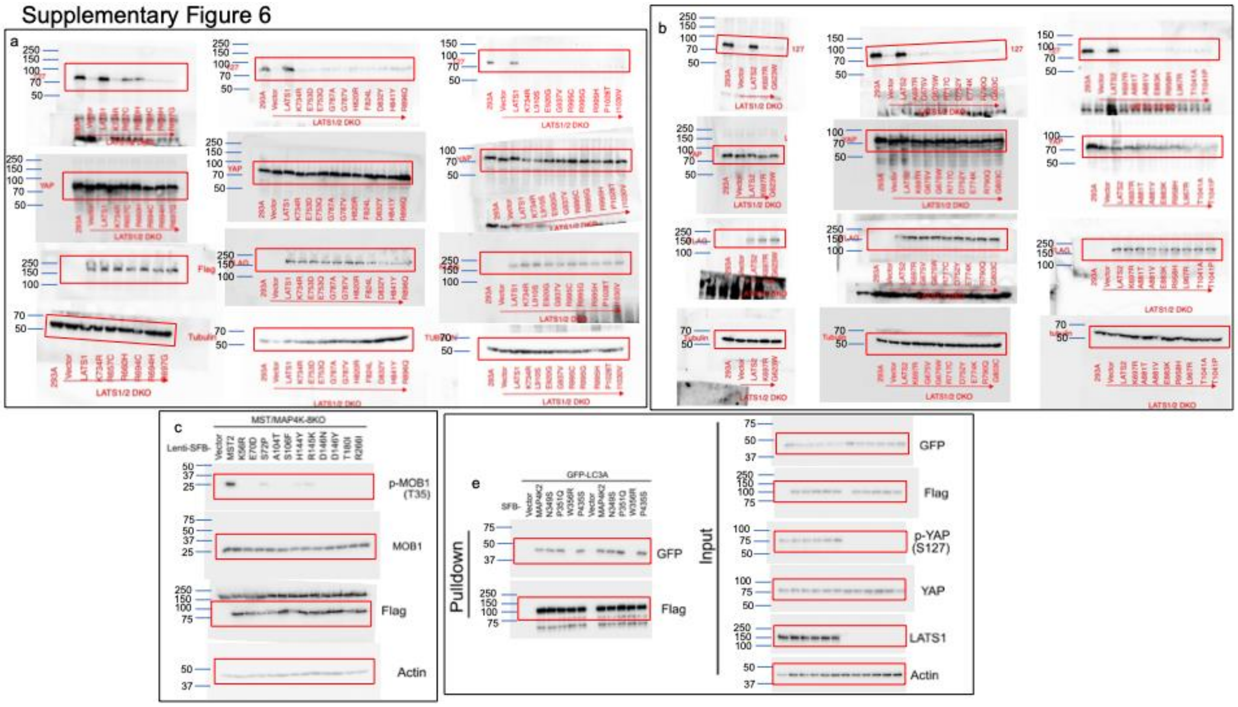

Supplementary Figure 12

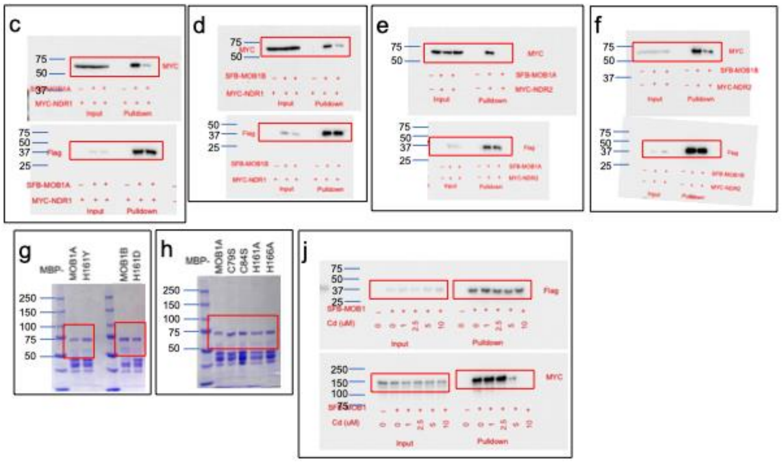

Supplementary Figure 14

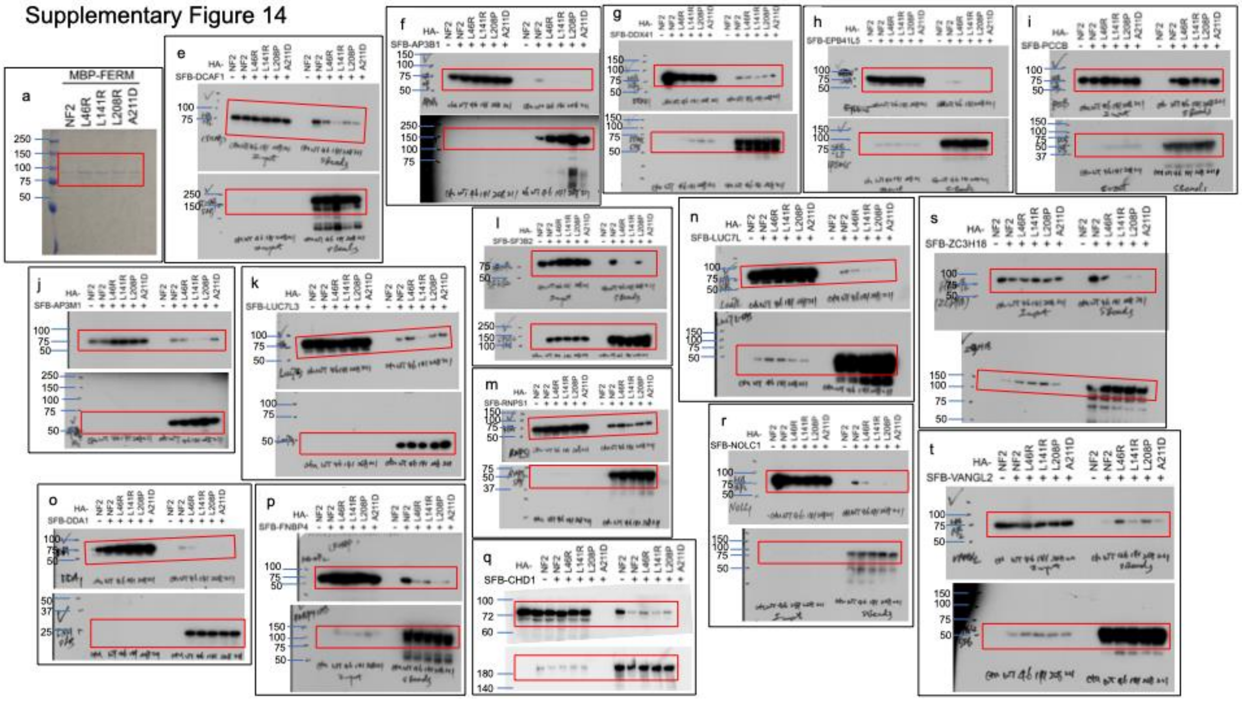

Supplement: Supplementary file 1 — Supplementary Information [file 41467_2024_54480_MOESM1_ESM.pdf]
