# Supplementary material for: Functional annotation of the Hippo pathway somatic mutations in human cancers
Source: Nat Commun. 2024 Nov 21;15:10106. doi: 10.1038/s41467-024-54480-y (PMC11582751; doi:10.1038/s41467-024-54480-y)
Supplement: Supplementary file 2 — Description of Additional Supplementary Files [file 41467_2024_54480_MOESM2_ESM.pdf]

Description of Additional Supplemental Files

Supplementary Data 1

Summary of Hippo signaling alterations in TCGA. Chi-squared test is used for the indicated analyses.

Supplementary Data 2

Summary of Hippo signaling somatic mutations in TCGA.

Supplementary Data 3

Summary of the Hippo pathway missense mutations used in this study. Chi-squared test is used for the indicated analyses.

Supplementary Data 4

Summary of the oncogenic alterations for Hippo signaling genes and control genes in TCGA.

Supplementary Data 5

Sequence information of the oligos used in this study.
