# Supplementary material for: Functional annotation of the Hippo pathway somatic mutations in human cancers
Source: Nat Commun. 2024 Nov 21;15:10106. doi: 10.1038/s41467-024-54480-y (PMC11582751; doi:10.1038/s41467-024-54480-y)
Supplement: Supplementary file 4 — Reporting Summary [file 41467_2024_54480_MOESM4_ESM.pdf]

nature portfolio

Corresponding author(s): Wenqi Wang

Last updated by author(s): Nov 5, 2024

Reporting Summary

Nature Portfolio wishes to improve the reproducibility of the work that we publish. This form provides structure for consistency and transparency in reporting. For further information on Nature Portfolio policies, see our Editorial Policies and the Editorial Policy Checklist.

Statistics

For all statistical analyses, confirm that the following items are present in the figure legend, table legend, main text, or Methods section.

n/a	Confirmed	
☐	☒	The exact sample size (*n*) for each experimental group/condition, given as a discrete number and unit of measurement
☐	☒	A statement on whether measurements were taken from distinct samples or whether the same sample was measured repeatedly
☐	☒	The statistical test(s) used AND whether they are one- or two-sided<br>*Only common tests should be described solely by name; describe more complex techniques in the Methods section.*
☒	☐	A description of all covariates tested
☒	☐	A description of any assumptions or corrections, such as tests of normality and adjustment for multiple comparisons
☐	☒	A full description of the statistical parameters including central tendency (e.g. means) or other basic estimates (e.g. regression coefficient) AND variation (e.g. standard deviation) or associated estimates of uncertainty (e.g. confidence intervals)
☐	☒	For null hypothesis testing, the test statistic (e.g. *F*, *t*, *r*) with confidence intervals, effect sizes, degrees of freedom and *P* value noted<br>*Give P values as exact values whenever suitable.*
☒	☐	For Bayesian analysis, information on the choice of priors and Markov chain Monte Carlo settings
☒	☐	For hierarchical and complex designs, identification of the appropriate level for tests and full reporting of outcomes
☒	☐	Estimates of effect sizes (e.g. Cohen's *d*, Pearson's *r*), indicating how they were calculated

Our web collection on statistics for biologists contains articles on many of the points above.

Software and code

Policy information about availability of computer code

Data collection	NIS-Elements BR 5.11.01 software was used for immunofluorescence data collection.<br>StepOne Software v2.3 was used for real-time PCR data collection.<br>Spectra Manager Ver.2 was used to collect Circular dichroism (CD) data.<br>ChemiDoc MP Imaging Systems and ImageLab 6.1 software was used to collect western blot data.
Data analysis	Microsoft Excel for Mac Version 16.67 was used for Student’s t-test (two sided) analysis.<br>The PMEMD program implemented in AMBER22 molecular dynamics suite was used for simulation analyses, CPPTRAJ was used to calculate the root-mean-square deviation (RMSD) value.

For manuscripts utilizing custom algorithms or software that are central to the research but not yet described in published literature, software must be made available to editors and reviewers. We strongly encourage code deposition in a community repository (e.g. GitHub). See the Nature Portfolio guidelines for submitting code & software for further information.

Data

Policy information about availability of data

All manuscripts must include a data availability statement. This statement should provide the following information, where applicable:

- Accession codes, unique identifiers, or web links for publicly available datasets
- A description of any restrictions on data availability
- For clinical datasets or third party data, please ensure that the statement adheres to our policy

The proteomic data generated in this study have been deposited in the ProteomeXchange Consortium database via the PRIDE partner repository with the dataset identifier PXD049472:
Project Name: Human Hippo cancer mutation proteins TAP-LC-MSMS; Project accession: PXD049472 (https://www.ebi.ac.uk/pride/archive/projects/PXD049472); Project DOI: 10.6019/PXD049472. Source data are provided with this paper.

Research involving human participants, their data, or biological material

Policy information about studies with human participants or human data. See also policy information about sex, gender (identity/presentation), and sexual orientation and race, ethnicity and racism.

Reporting on sex and gender	*Use the terms sex (biological attribute) and gender (shaped by social and cultural circumstances) carefully in order to avoid confusing both terms. Indicate if findings apply to only one sex or gender; describe whether sex and gender were considered in study design; whether sex and/or gender was determined based on self-reporting or assigned and methods used. Provide in the source data disaggregated sex and gender data, where this information has been collected, and if consent has been obtained for sharing of individual-level data; provide overall numbers in this Reporting Summary. Please state if this information has not been collected. Report sex- and gender-based analyses where performed, justify reasons for lack of sex- and gender-based analysis.*
Reporting on race, ethnicity, or other socially relevant groupings	*Please specify the socially constructed or socially relevant categorization variable(s) used in your manuscript and explain why they were used. Please note that such variables should not be used as proxies for other socially constructed/relevant variables (for example, race or ethnicity should not be used as a proxy for socioeconomic status). Provide clear definitions of the relevant terms used, how they were provided (by the participants/respondents, the researchers, or third parties), and the method(s) used to classify people into the different categories (e.g. self-report, census or administrative data, social media data, etc.) Please provide details about how you controlled for confounding variables in your analyses.*
Population characteristics	*Describe the covariate-relevant population characteristics of the human research participants (e.g. age, genotypic information, past and current diagnosis and treatment categories). If you filled out the behavioural & social sciences study design questions and have nothing to add here, write "See above."*
Recruitment	*Describe how participants were recruited. Outline any potential self-selection bias or other biases that may be present and how these are likely to impact results.*
Ethics oversight	*Identify the organization(s) that approved the study protocol.*

Note that full information on the approval of the study protocol must also be provided in the manuscript.

Field-specific reporting

Please select the one below that is the best fit for your research. If you are not sure, read the appropriate sections before making your selection.

☒ Life sciences ☐ Behavioural & social sciences ☐ Ecological, evolutionary & environmental sciences

For a reference copy of the document with all sections, see nature.com/documents/nr-reporting-summary-flat.pdf

Life sciences study design

All studies must disclose on these points even when the disclosure is negative.

Sample size	No statistical method was used to predetermine sample size. Sample size was determined based on our previous studies and common practice in the field.
Data exclusions	No samples or animals were excluded for the analyses in this study
Replication	Each experiment was repeated twice or more unless otherwise noted.
Randomization	As for the mouse experiments, we assigned the animals randomly to different groups.
Blinding	A laboratory technician was blinded to the group allocation during the animal experiments as well as the data analyses. As for other

Blinding	experiments, data collection and analysis were performed blindly.

Reporting for specific materials, systems and methods

We require information from authors about some types of materials, experimental systems and methods used in many studies. Here, indicate whether each material, system or method listed is relevant to your study. If you are not sure if a list item applies to your research, read the appropriate section before selecting a response.

Materials & experimental systems

n/a	Involved in the study	
☐	☒	Antibodies
☐	☒	Eukaryotic cell lines
☒	☐	Palaeontology and archaeology
☐	☒	Animals and other organisms
☒	☐	Clinical data
☒	☐	Dual use research of concern
☒	☐	Plants

Methods

n/a	Involved in the study	
☒	☐	ChIP-seq
☒	☐	Flow cytometry
☒	☐	MRI-based neuroimaging

Antibodies

Antibodies used

Sigma-Aldrich:
anti-alpha-tubulin (T6199-200UL, WB: 1:5000 dilution),
anti-Flag (M2)-peroxidase (HRP) (A8592-1MG, WB: 1:5000 dilution)
anti-Flag (M2) (F7425-.2MG, IF: 1:5000 dilution)
anti-beta-actin (A5441-100UL, 1:5000 dilution)

Santa Cruz Biotechnology:
anti-Myc (sc-40, WB: 1:500 dilution),
anti-YAP antibody (sc-101199, IF: 1:200 dilution)
anti-GFP (sc-390394, 1:1000 dilution)

BioLegend:
anti-hemagglutinin (HA) antibody (MMS-101P, WB: 1:3000 dilution)

Cell Signaling Technology:
anti-phospho-YAP (Ser127) (4911S, WB: 1:1000 dilution),
anti-phospho-LATS1 (Thr1079) (8654S, WB: 1:1000 dilution),
anti-LATS1 (3477S, WB: 1:1000 dilution, IP: 1:300 dilution),
anti-phospho-MST (Thr180/Thr183) (3681S, WB: 1:1000 dilution),
anti-MST1 (3682S, WB: 1:1000 dilution, IP: 1:300 dilution),
anti-phospho-MOB1 (Thr35) (8699S, WB: 1:1000 dilution),
anti-phospho-MOB1 (Thr12) (8843S, WB: 1:1000 dilution)
anti-MOB1 (3863S, WB: 1:2000 dilution),
anti-NF2 (12896S, WB: 1:2000 dilution),
anti-phospho-AKT (Ser473) (4060S, WB: 1:1000 dilution),
anti-AKT (4691S, WB: 1:2000 dilution),
anti-phospho-p44/42 MAPK (Erk1/2) (Thr202/Tyr204) (4370S, WB: 1:1000 dilution),
anti-p44/42 MAPK (Erk1/2) (9102S, WB: 1:2000 dilution),
anti-phospho-JNK (Thr183/Tyr185) (9251S, WB: 1:1000 dilution),
anti-JNK (9252S, WB: 1:2000 dilution),
anti-phospho-c-Jun (Ser63) (9261S, WB: 1:1000 dilution),
anti-c-Jun (9165T, WB: 1:2000 dilution),
anti-VANGL1 (14783S, WB: 1:1000 dilution)

Abcam:
anti-Thiophosphate ester antibody (ab92670, WB: 1:1000 dilution),

Kinexus:
anti-phospho-MAP4K2 (Ser170) antibody (AB-PK646, WB: 1:1000 dilution)

Homemade:
Anti-phospho-MAP4K3 (Ser170) (WB: 1:500 dilution)
anti-MBP (WB: 1:5000 dilution),
anti-GST (WB: 1:5000 dilution),
anti-YAP (WB: 1:500 dilution)

Validation

Most of the antibodies were validated in-house using knockout/knockdown cells by Western blot and/or immunofluorescence assays to evaluate their appropriate molecular weight bands or correct cellular localization, respectively. Phospho-antibodies were verified

using corresponding site mutation constructs and/or displaying the correct expression pattern between different treatments (e.g., kinase activating- or inactivating- stimuli, kinase KO cells, kinase inhibitors).

Eukaryotic cell lines

Policy information about cell lines and Sex and Gender in Research

Cell line source(s)	HEK293T (a female cell line, ATCC: CRL-3216) and MCF10A (a female cell line, ATCC: CRL-10317) were purchased from ATCC and kindly provided by Dr. Junjie Chen (MD Anderson Cancer Center). HEK293A (a female cell line, Thermo Fisher Scientific: R70507) was kindly provided by Dr. Jae-Il Park (MD Anderson Cancer Center). CAL-27 (a male cell line, ATCC: CRL-2095) and IOMM-Lee (a male cell line, ATCC: CRL-3370) were purchased from ATCC.
Authentication	No additional authentication was used for the cell lines.
Mycoplasma contamination	All the cell lines used in this study were tested negative by DAPI staining and PCR for mycoplasma.
Commonly misidentified lines (See ICLAC register)	None of the cell lines used in this study are listed in ICLAC.

Animals and other research organisms

Policy information about studies involving animals; ARRIVE guidelines recommended for reporting animal research, and Sex and Gender in Research

Laboratory animals	Athymic nude (nu/nu) mouse strain was used for the xenograft tumor assays in this study. All the nude mice were purchased from Jackson Laboratory (002019) and kept in a pathogen-free environment in ULAR Facility at UCI under 14-hour light/10-hour dark cycle with temperature of 75°F and 60% humidity.
Wild animals	No wild animals were used in this study.
Reporting on sex	Eight-week-old female nude mice were used for orthotopic xenograft tumor assay. Four-week-old female nude mice were used for xenograft tumor study.
Field-collected samples	No filed-collected samples were used in this study.
Ethics oversight	All the tumor assays were followed with institutional guidelines, approved by the Institutional Animal Care and Use Committee (IACUC; protocol number AUP-19-112) of the University of California, Irvine, and performed under veterinary supervision.

Note that full information on the approval of the study protocol must also be provided in the manuscript.

Plants

Seed stocks	*Report on the source of all seed stocks or other plant material used. If applicable, state the seed stock centre and catalogue number. If plant specimens were collected from the field, describe the collection location, date and sampling procedures.*
Novel plant genotypes	*Describe the methods by which all novel plant genotypes were produced. This includes those generated by transgenic approaches, gene editing, chemical/radiation-based mutagenesis and hybridization. For transgenic lines, describe the transformation method, the number of independent lines analyzed and the generation upon which experiments were performed. For gene-edited lines, describe the editor used, the endogenous sequence targeted for editing, the targeting guide RNA sequence (if applicable) and how the editor was applied.*
Authentication	*Describe any authentication procedures for each seed stock used or novel genotype generated. Describe any experiments used to assess the effect of a mutation and, where applicable, how potential secondary effects (e.g. second site T-DNA insertions, mosiacism, off-target gene editing) were examined.*
